# Hardware Infection From Surgical Stabilization of Rib Fractures Is Lower Than Previously Reported

**DOI:** 10.7759/cureus.35732

**Published:** 2023-03-03

**Authors:** Zachary M Bauman, Krysta Sutyak, Trevor A Daubert, Hason Khan, Tylor King, Kevin Cahoy, Meghana Kashyap, Emily Cantrell, Charity Evans, Adam Kaye

**Affiliations:** 1 Surgery, University of Nebraska Medical Center, Omaha, USA; 2 Surgery, Overland Park Regional Medical Center, Kansas City, USA

**Keywords:** chest wall injury, non-union, rib fixation, surgical stabilization of rib fractures, hardware infection

## Abstract

Introduction

Surgical stabilization of rib fractures (SSRF) is an emerging therapy for the treatment of patients with traumatic rib fractures. Despite the demonstrated benefits of SSRF, there remains a paucity of literature regarding the complications from SSRF, especially those related to hardware infection. Currently, literature quotes hardware infection rates as high as 4%. We hypothesize that the hardware infection rate is much lower than currently published.

Methods

This is an IRB-approved, four-year multicenter descriptive review of prospectively collected data from January 2016 to June 2022. All patients undergoing SSRF were included in the study. Exclusion criteria included those patients less that 18 years of age. Basic demographics were obtained: age, gender, Injury Severity Score (ISS), Abbreviate Injury Scale-chest (AIS-chest), flail chest (yes/no), delayed SSRF more than two weeks (yes/no), number of patients with a pre-SSRF chest tube, and number of ribs fixated. Primary outcome was hardware infection. Secondary outcomes included mortality rate and hospital length of stay (HLOS). Basic descriptive statistics were utilized for analysis.

Results

A total of 453 patients met criteria for inclusion in the study. Mean age was 63 ± 15.2 years and 71% were male. Mean ISS was 17.3 ± 8.5 with a mean AIS-chest of 3.2 ± 0.5. Flail chest (three consecutive ribs with two or more fractures on each rib) accounted for 32% of patients. Forty-two patients (9.3%) underwent delayed SSRF. The average number of ribs stabilized was 4.75 ± 0.71. When analyzing the primary outcome, only two patients (0.4%) developed a hardware infection requiring reoperation to remove the plates. Overall HLOS was 10.5 ± 6.8 days. Five patients suffered a mortality (1.1%), all five with ISS scores higher than 15 suggesting significant polytrauma.

Conclusion

This is the largest case series to date examining SSRF hardware infection. The incidence of SSRF hardware infection is very low (<0.5%), much less than quoted in current literature. Overall, SSRF is a safe procedure with low morbidity and mortality.

## Introduction

Rib fractures are a major consideration in trauma patients - prevalent in up to 10% of all trauma patients and one of the most common injuries in blunt trauma [1-6]. There is an abundance of research that concludes patients with rib fractures, either isolated or as a component of polytrauma, have higher levels of morbidity and mortality than patients without rib fractures [1-8]. The presence of eight or more rib fractures alone results in mortality rates to rise to almost 35% and one-third of patients with rib fractures, regardless of their other injuries, will require discharge to a rehabilitation center [9,10]. Surgical Stabilization of Rib Fractures (SSRF) is a relatively new, emerging, and evolving therapy for the treatment of traumatic rib fractures. The benefits are well-publicized and include decreased pain and narcotic utilization, decreased incidence of pneumonia and respiratory failure, decreased need for a tracheostomy, decreased intensive care unit (ICU) length of stay (LOS), decreased hospital LOS (HLOS), and decreased mortality [4,7-11]. Despite the demonstrated benefits of SSRF, there still remains hesitancy to utilize this treatment modality, likely due to a paucity of literature regarding SSRF complications, especially those related to hardware infection.

The surgical community has been treating hardware infections for decades in many specialties where hardware implants are routinely utilized [12,13]. Infections can range from mild to severe, local to systemic, and can lead to non-union or malunion of the original boney injury [13,14]. Traditionally, infected hardware requires treatment with systemic antibiotics, implantation of antibiotic beads, and in severe cases, removal of the infected hardware with multiple secondary interventions [13,14]. This presents a difficult and complicated treatment course with overall significantly increased morbidity and cost [13,14]. Looking specifically at the literature regarding SSRF, anywhere between a 0-10% infection rate is quoted with the most recent and specific data suggesting 4% [13,15-17]. In contrast, when we look at rates of hardware infection for other types of closed fractures, they are around 0.5-2% [13,18-20].

Given the significance of complications and resultant interventions that can result from hardware infection, we wanted to further examine this problem in the SSRF patient population. The objective of this study was to further assess hardware infection rates in this patient population through a multi-institutional approach. We hypothesize that hardware infection rates in SSRF patients are lower than previously reported.

## Materials and methods

This was an Institutional Review Board-approved, multicenter descriptive review of prospectively collected data from January 2016 through June 2022. Trauma centers involved were at least an American College of Surgeons Level II Trauma Center or higher. This includes a Level I academic trauma center and a Level II private trauma center. Both are located in very urban areas and admit over 2,000 trauma patients annually. All patients who underwent SSRF during this study period were included in this study. This included patients that underwent SSRF acutely during their index hospitalization for their traumatic rib fractures or those patients who underwent delayed rib fixation (defined as any time greater than two weeks from acquiring their rib fractures) for fracture non-union. All patients undergoing SSRF had closed fractures. In the acute setting, all attempts were made to perform SSRF within 72 hours of injury although this was not always possible given concomitant patient injuries and pathophysiology. Patients were excluded from the study if they were younger than 18 years of age. Basic demographics were obtained including age, gender, Injury Severity Score (ISS), Abbreviate Injury Scale-chest (AIS-chest), presence of flail chest, whether the patient required chest tube placement at admission (prior to SSRF), and the average number of ribs fixed per patient. In addition, patient comorbidities as well as body mass index were obtained and reported.

The primary outcome of interest was the development of hardware infection. Hardware infection was defined by any chest wall incision related to SSRF that developed erythema, heat, or presumed purulent discharge requiring opening of the incision as well as prophylactic antibiotics for a presumed infection. Furthermore, any surgical site in which an abscess developed and was confirmed via cultures requiring antibiotic treatment, or any SSRF surgical field that required removal of the hardware due to obvious or presumed infection was classified as hardware infection. Secondary outcomes included mortality rate and HLOS. Given this was a descriptive analysis, basic descriptive statistics were utilized for analysis.

All SSRF procedures were completed by trauma surgeons using a muscle-sparing technique, which has now been recognized as the standard of care for this procedure given it minimizes additional damage to the chest wall soft tissue [11,21]. Titanium plates were utilized for all procedures. It was left to the discretion of the treating surgeon as to the brand of titanium plate and whether intrathoracic or extrathoracic plates were utilized. Incisions are kept as small as possible to further minimize post-operative pain. Hemostasis was meticulously addressed and incisions were closed in a series of layers using vicryl suture to reapproximate the muscle layers as well as fascia, followed by a running subcuticular stitch for the skin layer or staples. Sterile dressings were applied to all incisions and left in place for two days following surgery. All patients were placed on similar multimodal pain regimens before and after surgery, which were tailored to individual patient needs. Furthermore, all the patients received a single dose of pre-operative antibiotics within one hour of incision time. The antibiotic of choice was left to the discretion of the operating surgeon and based on individual patient allergies. However, all antibiotics utilized were recognized as appropriate surgical prophylactic antibiotics for chest wall surgery. Furthermore, all study patients underwent chest cavity washout with sterile normal saline followed by placement of a sterile chest tube while still in the operating room as a routine part of the surgery. The removal of the chest tube was left to the discretion of the treating surgeon and based on current published practice management principles. Of note, patients undergoing SSRF typically have their chest tube for one to two days postoperatively. All patients who developed a concomitant infection (ie, urinary tract infection, etc) during their hospitalization were treated appropriately. Lastly, SSRF procedures ranged anywhere from 1.5 to 6.5 hours and all patients who required a re-dose of antibiotics received it appropriately based on the antibiotic given prior to anesthesia induction and its half-life.

## Results

A total of 453 patients over this time period qualified for inclusion in this study for a total of 2,153 ribs that were stabilized with titanium plates. An average of 4.75 ± 0.71 ribs were plated per patient. The mean age for all study participants was 63 ± 15.2 years. Males made up the majority of the study population at 71% prevalence. The mean ISS was 17.3 ± 8.5 with a mean AIS-chest of 3.2 ± 0.5. Flail chest (three or more consecutive ribs broken in two or more locations) accounted for 32% of the SSRF population. Forty-two patients underwent delayed SSRF, accounting for 9.3% of the study population. Approximately half (48%) of the study population had a chest tube placed prior to SSRF and 77.9% of all SSRF patients had concomitant cryoablation of their intercostal nerves (Table 1).

**Table 1:** Patient Demographics SD = standard deviation; ISS = Injury Severity Score; AIS = Abbreviated Injury Severity Scale; SSRF = surgical stabilization of rib fractures

Characteristic	Results
Total Number of Patients	453
Total Number of Ribs Plated	2,153
Number Ribs Plated/Patient, mean (SD)	4.75 (0.71)
Age (SD)	63 (15.2)
Gender, male (%)	321 (71)
ISS, mean (SD)	17.3 (8.5)
AIS – chest, mean (SD)	3.2 (0.5)
Delayed fixation (%)	42 (9.3)
Concomitant Cryoablation Intercostal Nerves (%)	353 (77.9)
Patients with Pre-SSRF Chest Tube (%)	218 (48.1)
Combined SSRF with Sternal Fixation (%)	19 (4.2)
Patients with Intrathoracic Plates (%)	42 (9.3)
Comorbidites	
Current Smoker, N (%)	103 (22.7)
Chronic Pulmonary Obstructive Disease, N (%)	37 (8.3)
Osteoporosis, N (%)	18 (4.0)
Cardiac Disease, N (%)	46 (10.2)
Previous Cardiac Surgery, N (%)	15 (3.3)
Hypertension, N (%)	193 (42.6)
Diabetes Mellitus, N (%)	74 (16.3)
Mental Health Disorder, N (%)	72 (15.9)
Hepatits C, N (%)	11 (2.4)
Alcohol/Substance Abuse, N (%)	73 (16.1)
Other, N (%)	113 (24.9)
Antiplatelets/Anticoagulation, N (%)	91 (20.1)
Steroids, N (%)	18 (4.0)
Body Mass Index, mean (SD)	31.1 (6.4)
Body Mass Index >35, N (%)	79 (17.9)

When analyzing the outcomes of interest, only two patients (0.4%) developed a hardware infection based on the above-described definition. Neither patient developed their infection until after 30 days from their operation and both required hardware removal for clearance of the infection. Furthermore, one of the two patients that developed infected hardware was a redo of a delayed SSRF case resulting from secondary trauma to the chest. A summary of each hardware infection patient can be found in Table 2. Overall HLOS was 10.5 ± 6.8 days. Five total patients in this study population suffered a 30-day mortality (1.1%), none of which were directly related to the hardware infection from the SSRF.

**Table 2:** Infected Hardware Patients PMH = past medical history; SSRF = surgical stabilization of rib fractures

Age	Mechanism	SSRF Details	Timing of Hardware Infection	Treatment
57	Fall from bike	Non-union of right later fractures 8-10. Repaired 1.5 years after injury. PMH: Asthma, attention deficit disorder	79 days	Plate removal with wound vac placement
63	Tree branch fell on chest	Repaired on HD #3. SSRF of left anterior (involved costocartilage) rib fractures 3 – 6. PMH: Coronary artery disease, pulmonary fibrosis, anxiety, hyperlipidemia, anxiety, hypothyroidism, tobacco abuse	51 days	Plate removal with rib/sternal debridement. Wound vac placement

## Discussion

Hardware infection related to SSRF is a rare complication. To the authors' knowledge, this is the largest case series and the only multi-institutional study reporting that the rate of hardware infection from SSRF remains relatively low. In fact, this study demonstrates the lowest hardware infection rate (0.4%) for SSRF compared to all previous reports in the current literature. This is despite 32% of the study population having significant chest wall trauma with defined flail chest and approximately half the study population (48%) having a chest tube placed prior to SSRF. Furthermore, this study points out a very low 30-day mortality rate associated with SSRF (1.1%), with no mortality directly related to hardware infection.

Hardware infection related to SSRF is a complication that must be discussed with the patient prior to surgery, however, the incidence with which this happens may not be as high as previously reported. A previous study from 2016 by Thiels et al. suggested an overall hardware infection rate of 4.1% at an average of 12 days postoperatively [13]. This study only included 122 patients that underwent SSRF. Our study demonstrates a much lower hardware infection rate with almost four times more patients. One possible explanation for this is the surgical technique for SSRF has evolved substantially over the past decade [11]. With the development of the muscle-sparing technique as well as utilization of incisions uniquely placed over the rib fracture patterns, smaller incisions and less chest wall tissue damage from the surgery itself have been the result [11,21]. This has likely contributed to decreased rates of hardware infection. Furthermore, with the advancement of the technique to a more minimally invasive, intrathoracic thoracoscopic approach, again the size of the incisions has decreased resulting in decreased chest wall tissue damage and less chance for surgical site infections [22].

Other studies have been completed looking at hardware infection. A large systemic review of 1,952 patients undergoing SSRF in 48 different studies reported a hardware infection rate of 2.2% [23]. Junker et al. reported an infection rate of 3.5% among 285 patients who underwent SSRF [24]. Furthermore, a study by Prins et al., analyzing 228 patients over a 10-year period who underwent SSRF, showed a hardware infection rate of 1.8% in which all patients required hardware removal [25]. Comparatively, our study demonstrated an even lower risk of hardware infection at 0.4% with over double the number of patients compared to the Prins et al. study. Similarly, the two infected hardware patients in our study did require a second surgery to remove their plates, however, they did well post-operatively and were able to discharge from the hospital. It is important to point out that of the two infected hardware cases in our study, only one of the patients developed their hardware infection after an acute SSRF (fixation of the rib fractures during the index hospitalization for the patient’s traumatic rib fractures), which is what the aforementioned studies were focusing on. The second patient in our study underwent a delayed fixation (1.5 years after his injury) due to a non-union of his rib fractures. He subsequently had recurrent trauma to his chest wall prior to the ribs healing resulting in a fractured plate and the need to re-operate to stabilize that rib fracture again. That is when he developed his hardware infection, after this re-operation. Therefore, our study shows a hardware infection rate in the acute setting of only 0.2%.

Despite the low hardware infection rate demonstrated in this study (and the literature), the treatment has historically been systemic antibiotics and surgery requiring hardware removal [13,24-26]. Often the wound care can be tedious for these infections, requiring additional bone and soft tissue debridement, and the rib fractures often demonstrate a non-union at the time of hardware explanation, which was the case in our study [13,24,25]. A recent study by Junker et al. utilized a management strategy becoming more common in the field of orthopedic infected joints: antibiotic-impregnated cement beads [24,27,28]. They placed vancomycin and gentamicin cement beads in nine infected SSRF surgical fields and another eight SSRF surgical fields prophylactically for high-risk patients [24]. All 17 patients demonstrated bony union by four months postoperatively [24]. No infections occurred for patients in which the beads were placed prophylactically, and all nine infected fields were salvaged from the infection until the fractures had healed [24]. Although we did not do this for our study, this technique likely should be considered for future hardware infections, especially if the fractures demonstrate they have not healed at the time the infection occurs.

There are several limitations to this study. First, this was an observational review of prospectively collective data. Prospective, randomized controlled studies are always preferred as there is inherent selection bias associated with this type of review. This could affect the overall results as the selection bias may have favored non-operative management for patients with more comorbidities (ie. diabetes, chronic steroid use, etc) that could impair healing. Attempts were made to mitigate this by including more than one center in this review, and although all centers involved do not utilize these comorbidities as contraindications for surgery, a prospective trial can reduce this bias even further. Second, this study only focused on hardware infection rates and not the various costs or patient implications associated with them. The patients in this study were managed with traditional surgical intervention, and although this is not necessarily a morbid procedure, it is not without its own complications and risks. Not to mention, wound management can be very challenging and time-consuming for the patient. Although we did present an alternative method (and potentially less morbid/invasive) for managing hardware infections utilizing antibiotic beads, future studies should focus on management of these infections as well as associated healthcare costs. Lastly, this was a descriptive analysis only. Given the low number of hardware infections, we were unable to run a multi-linear regression analysis to see if there are any comorbidities, patient characteristics, etc. that are specifically associated with a higher risk of developing a hardware infection. This would be very helpful for future studies to try and better predict who is at a higher risk for developing these infections.

## Conclusions

Hardware infection after SSRF is rare. The traditional management of these patients is systemic antibiotics and hardware removal with debridement and local wound care as needed. However, with more evidence coming out supporting the use of antibiotic-infused cement beads for hardware infection situations, this should be considered as a treatment option for these patients to potentially cut down on patient morbidity, specifically rib fracture non-union. Discussions should be held with patients prior to SSRF to assure they are aware of the risk of hardware infection, however they should be reassured the risk is extremely low. Although our patients had positive outcomes after their hardware infection despite rib fracture non-union, future studies should focus on patient costs related to SSRF hardware infection as well as patient indicators that would make them higher risk for developing a hardware infection. 
